# Successful coil occlusion of intracranial aneurysm in a child with STAT3 hyper IgE syndrome

**DOI:** 10.70962/jhi.20250028

**Published:** 2025-06-02

**Authors:** Archan Sil, Chirag K. Ahuja, Pandiarajan Vignesh, Amit Rawat, Biman Saikia

**Affiliations:** 1 Allergy Immunology Unit, Department of Pediatrics, Advanced Pediatrics Centre, Postgraduate Institute of Medical Education and Research, Chandigarh, India; 2 Department of Radiodiagnosis and Imaging, Postgraduate Institute of Medical Education and Research, Chandigarh, India; 3 Allergy Immunology Unit, Department of Pediatrics, Advanced Pediatrics Centre, Postgraduate Institute of Medical Education and Research, Chandigarh, India; 4 Allergy Immunology Unit, Department of Pediatrics, Advanced Pediatrics Centre, Postgraduate Institute of Medical Education and Research, Chandigarh, India; 5 Department of Immunopathology, Postgraduate Institute of Medical Education and Research, Chandigarh, India

## Abstract

A novel heterozygous STAT3 variant (p.Phe710Ser) was identified in an adolescent with hyper IgE syndrome and an intracranial aneurysm. Successful nonsurgical coil occlusion underscores the importance of vascular screening and the feasibility of interventional radiology in HIES.

## Teaser

Hyperimmunoglobulin E (hyper IgE) syndrome (HIES) is a rare form of inborn errors of immunity. We report an unusual presentation of intracranial aneurysm in an adolescent girl with AD-HIES. The child was managed successfully by coil occlusion of the intracranial aneurysm.

## Introduction

Hyperimmunoglobulin E (hyper IgE) syndrome (HIES) is a rare form of inborn errors of immunity (IEI), characterized by recurrent cold abscesses (predominantly staphylococcal), pulmonary infections, eczema, and elevated IgE levels. This entity was initially described as Job’s syndrome as its classical presentation of multiple cold abscesses had resemblance with the biblical character “Job.” Subsequently, heterozygous dominant-negative mutation in the *STAT3* gene on chromosome 17q21 was found to be associated with HIES. Connective tissue abnormalities like scoliosis, hyperextensibility of joints, bone fracture, retention of primary teeth, and coarse facial features are frequently reported in STAT3 HIES apart from recurrent infections. A National Institutes of Health (NIH) scoring system was developed in 1999 to facilitate identification of HIES in the clinics. Various vascular and brain abnormalities have been described in patients with HIES, including white matter hyperintensities, lacunar infarcts, ectasia, stenosis, and aneurysm (1). Herein, we report an unusual presentation of intracranial aneurysm in an adolescent girl with autosomal dominant (AD) HIES.

## Case report

An 11-year-old girl presented with multiple cold abscesses over the scalp and bilateral parotid region and meningitis. Blood and pus cultures grew methicillin-sensitive *Staphylococcus aureus*. Computed tomography (CT) of the chest showed pneumonic consolidation with pneumatocele. She was treated with intravenous ceftriaxone and cloxacillin. Based on clinical presentation, elevated serum IgE level (25,000 IU/ml), and low Th17 lymphocytes, a provisional diagnosis of AD HIES was made [NIH score-47] (Table S1). In-house–targeted next-generation sequencing using a targeted panel for IEI showed a novel heterozygous missense variant [c.2129T>C; p.Phe710Ser] at exon 22 of the *STAT3* gene. The amino acid position (F710) is situated in the trans-activation domain of the STAT3 protein and is highly conserved across species. The variant F710S is confirmed to be de novo in the child, has a CADD score of 32, a Polyphen score of 0.967 (probably damaging), and a mistic value of 0.98 (damaging).

**Figure 1.**
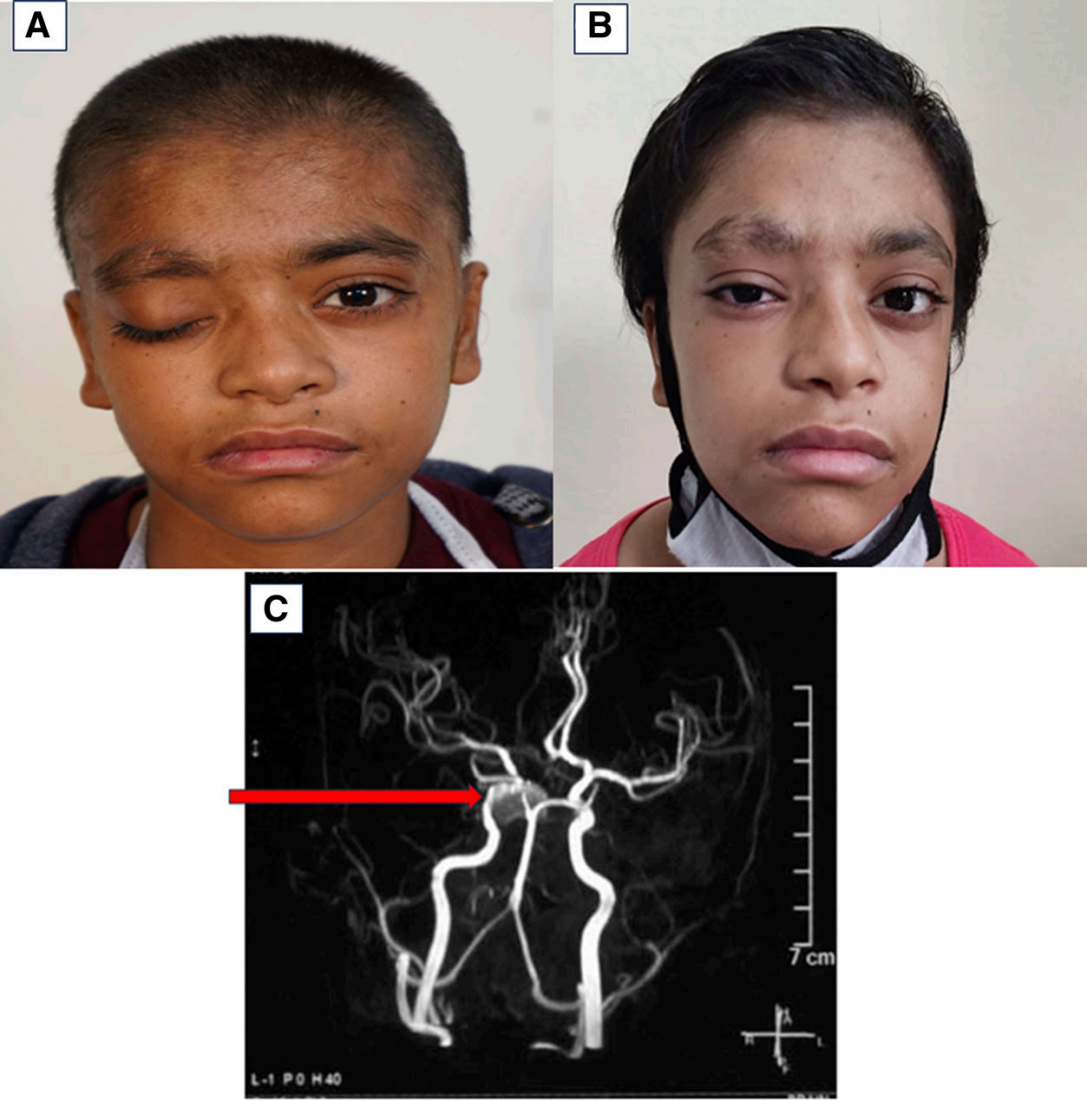
**Clinical and radiological findings in our patient with hyper-IgE syndrome. (A)** Right-sided ptosis before intervention. **(B)** Partial recovery of right-sided ptosis after coil occlusion of aneurysm in petro-cavernous part of ICA. **(C)** MR angiography showing aneurysm in the intracavernous part of right ICA.

On the seventh day of the hospital stay, the child developed sudden onset of ptosis with diplopia of the right eye. She also had repeated episodes of nausea and vomiting. On examination, there was diminished sensation on the right side of the forehead and cheek with drooping of the right eyelid (Fig. 1). Downward and outward deviation of the right eye was also noted. While the right sided pupil was dilated and sluggishly reacting to light, the size and light reaction of the left-sided pupil were normal. Findings were suggestive of right-sided third, fourth, and fifth cranial nerve palsy. Magnetic resonance (MR) imaging and MR angiography were done that showed an aneurysm in the intracavernous part of the right internal carotid artery (ICA) (Fig. 1).

CT-guided angiography was done to delineate the morphology of the intracranial aneurysm. The child underwent percutaneous (through the right femoral artery) fluoroscopic coil occlusion of the right petro-cavernous part of the ICA aneurysm under interventional radiology without any complication. The postoperative period was uneventful. There was subsidence of vomiting and headache following intervention. Although ptosis persisted in the immediate postoperative period, there was gradual recovery over the next few months (Fig. 1). The child was kept on follow-up with monthly intravenous immunoglobulin replacement and co-trimoxazole prophylaxis. At the 1-year follow-up, she is doing well with no recurrence of infection, and there was a significant reduction in eczematous lesions.

## Discussion

Vascular abnormalities in patients with AD HIES can involve arteries in the brain, coronaries, and peripheral arteries. Although the exact etiology of vascular malformations in HIES is not known, there are several explanations for aneurysm formation. Weakening of the vessel wall can be due to infections (mycotic), eosinophilia, autoimmune vasculitis, or genetic factors. Defective *STAT3* signaling plays an important role in determining the vascular phenotype of the patient (1). Fragility of the internal elastic lamina can be explained by defective collagen formation due to genetic defects. Defective angiogenesis and extracellular matrix formation have been mentioned as the contributing factors for the development of coronary artery aneurysms in patients with AD HIES (2). Chandesris et al. documented markedly reduced intima-media thickness as a cause of increased circumferential wall stress, which ultimately leads to arterial dilation (1). A detailed macroscopic and microscopic description of vasculopathy has been published in another report of an adult female with STAT3 deficiency (3). Elastase produced by fungus like *Aspergillus* has also been postulated as an important cause of vessel wall abnormality in patients with HIES (1, 4).

While there are few instances of cerebral aneurysms in adult patients with AD HIES in English literature, such reports are extremely rare in pediatric patients (4). Presentation in adults varied from headache (subarachnoid hemorrhage) to right-sided hemiparesis and loss of consciousness. The mode of diagnosis was CT or MR angiography, and they were mostly managed by interventions like clipping, coil insertion, or surgical ligation (4).

The index child presented with headache, vomiting, ptosis, and external ophthalmoplegia, suggestive of multiple cranial nerve (third, fourth, and sixth) involvement. This unique presentation prompted us to do neurological imaging (CT followed by MR angiography), which ultimately clinched the diagnosis of intracranial aneurysm. Prompt intervention by clipping of the aneurysm led to resolution of the symptoms. To the best of our knowledge, this is the first report of successful nonsurgical coil occlusion of a cerebral artery aneurysm in a child with STAT3 HIES. Kim et al. described a similar case of intracranial aneurysm in a 12-year-old boy with *STAT3* mutation (5). The child presented with a history of frequent ear discharge since early childhood. His family history was suggestive, with his father and two siblings having recurrent infections. At 12 years of age, he was diagnosed with a 2.5 × 2.4-cm aneurysm involving the petrous part of the right ICA while being evaluated for headache and diplopia. He was successfully managed by surgical ligation of the same (5).

## Conclusion

Management of cerebrovascular aneurysms is challenging in HIES and may require surgical or nonsurgical interventions to relieve symptoms. Headache in any patient of HIES warrants thorough neurological examination, including neuroimaging by either CT or MRI scan of the brain. Any abnormality of suspected vascular origin should be followed by CT or MR angiography. Timely diagnosis and prompt management of cerebral aneurysms can prevent dreadful complications in patients with HIES.

## Online supplemental material

Table S1 shows the NIH score for hyper IgE syndrome of the child.

## Supplementary Material

Table S1 shows the NIH score for hyper IgE syndrome of the child.

## Data Availability

The datasets generated during and/or analyzed during the current study are available from the corresponding author on reasonable request.
