# Supplementary material for: Successful coil occlusion of intracranial aneurysm in a child with STAT3 hyper IgE syndrome
Source: J Hum Immun. 2025 Jun 2;1(2):e20250028. doi: 10.70962/jhi.20250028 (PMC13177796; doi:10.70962/jhi.20250028)
Supplement: Table S1 — shows the NIH score for hyper IgE syndrome of the child. [file jhi_20250028_tables1.docx]

**Supplementary Table 1-** NIH score for hyper IgE syndrome of the child

| **CLINICAL FINDINGS** | **Values for the index child (POINTS)** |
| --- | --- |
| Highest serum-IgE level (IU/ml) | >2000 **(10)** |
| Skin abscesses | 4 **(4)** |
| Pneumonia (episodes over lifetime) | 1 **(2)** |
| Parenchymal lung anomalies | Pneumatocele **(8)** |
| Retained primary teeth | >3 **(8)** |
| Scoliosis, maximum curvature | <10° **(0)** |
| Fractures with minor trauma | None **(0)** |
| Highest eosinophil count (cells/ml) | >800 **(6)** |
| Characteristic face | Mildly present **(2)** |
| Midline anomaly | Absent **(0)** |
| Newborn rash | Present **(4)** |
| Eczema (worst stage) | Moderate **(2)** |
| Upper respiratory infections per year | 3 **(1)** |
| Candidiasis | None **(0)** |
| Other serious infections | None **(0)** |
| Fatal infection | Absent **(0)** |
| Hyperextensibility | Absent **(0)** |
| Lymphoma | Absent **(0)** |
| Increased nasal width | <1 SD **(0)** |
| High palate | Absent **(0)** |
| Young-age correction | >5 years **(0)** |
| **TOTAL SCORE** | **47** |
